# Improving HIV service delivery for people who inject drugs in Kazakhstan: study protocol for the Bridge stepped-wedge trial

**DOI:** 10.1186/s13012-019-0909-z

**Published:** 2019-06-14

**Authors:** Tara McCrimmon, Louisa Gilbert, Timothy Hunt, Assel Terlikbayeva, Elwin Wu, Meruyert Darisheva, Sholpan Primbetova, Azamat Kuskulov, Alissa Davis, Anindita Dasgupta, Bruce R. Schackman, Lisa R. Metsch, Daniel J. Feaster, Baurzhan Baiserkin, Nabila El-Bassel

**Affiliations:** 1 Global Health Research Center of Central Asia, Almaty, Kazakhstan; 2 Columbia University School of Social Work, 1255 Amsterdam Ave., New York, NY 10027 USA; 3 Weill Cornell Medical College, New York, NY USA; 4 Columbia University Mailman School of Public Health, New York, NY USA; 5 University of Miami Miller School of Medicine, Miami, FL USA; 6 The Kazakh Scientific Center of Dermatology and Infectious Diseases, Almaty, Kazakhstan

**Keywords:** HIV, Harm reduction, People who inject drugs (PWID)

## Abstract

**Background:**

People who inject drugs (PWID) in Kazakhstan face many barriers to HIV testing as well as to accessing HIV care, to retention in HIV care, and to initiating and adhering to anti-retroviral treatment (ART). Needle and syringe programs (NSPs) are an opportune setting for integrated interventions to link PWID to HIV care.

**Methods:**

This Hybrid Type II study employs a stepped-wedge design to evaluate both effectiveness and implementation outcomes of Bridge, an intervention to identify, test, and link HIV-positive PWID to HIV care. The study is conducted at 24 NSPs in three different regions of Kazakhstan, to assess outcomes on the individual, organizational, and policy levels.

**Discussion:**

This trial responds to an identified need for new models of HIV service delivery for PWID through harm reduction settings.

**Trial registration:**

NCT02796027 on June 10, 2016.

Contributions to the literature

- This paper describes the Bridge study, an implementation study to improve access to HIV testing and linkage to care among people who inject drugs (PWID) in Kazakhstan. This is the first HIV implementation study among PWID conducted in Central Asia.
- Provides an example of a stepped-wedge design and robust multi-level assessments to evaluate effectiveness and implementation outcomes of Bridge.
- Can serve as a model of a differentiation of care approach that utilizes harm reduction settings for HIV testing, linkage, and retention in care.

## Background

Injection drug use remains a large contributor to the HIV epidemic in Kazakhstan, a country with an estimated 127,800 people who inject drugs (PWID) [1]. The prevalence of HIV among Kazakhstan’s PWID is higher than any other key population at 9.2% (compared to 1.9% for sex workers, 6.1% for men who have sex with men, and 3.5% for prisoners) [2]. Parenteral transmission of HIV through injection drug use accounts for 54% of the approximately 26,887 registered cases of HIV in Kazakhstan’s history [3]. Furthermore, sexual transmission of HIV often occurs among the sexual partners of injection drug users [3], and one study in Kazakhstan found an HIV prevalence of 10.4% among non-PWID partners of PWID [4].

**Fig. 1:**
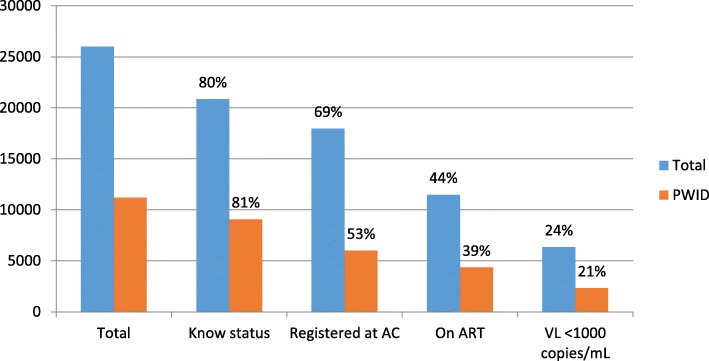
HIV care cascade among total population of people living with HIV (PLHIV) and among people who inject drugs (PWID) in Kazakhstan [5]

Large gaps in the continuum of HIV care for PWID have intensified the epidemic among this community, as indicated in Fig. 1. In 2017, Kazakhstan’s Republican AIDS Center estimated that there were 11,207 HIV-positive PWID in the country, with 9072 (80.9%) knowing their status. However, fewer than half (4340, 38.7%) received antiretroviral treatment (ART) and only 2318 (20.6%) had a viral load below 1000 copies/mL (the threshold in Kazakhstan for viral suppression) [5]. This drop is consistent with research worldwide [6–9]. HIV-positive PWID who remain untreated face increased HIV-related morbidity and mortality and continue to spread the virus through sexual and injection drug use networks [10].

Communities and medical providers often believe that PWID will not adhere to treatment and therefore are at higher risk of developing drug resistance [11, 12]. However, research suggests that they benefit from ART just as much as those who do not inject drugs do [12–15]. Suppressing viral load among PWID decreases the spread of HIV; furthermore, treatment initiation among PWID is associated with subsequent reductions in unprotected sex and unsafe injections [10]. A longitudinal study showed that expansion of ART within a PWID community in Vancouver, Canada, significantly decreased HIV incidence within the same community [16]. It is therefore critical to identify and link HIV-positive PWID to care, with the ultimate aim of initiating ART and achieving viral suppression, as well as linking them to harm reduction programs and other services addressing common comorbidities such as drug addiction, sexually transmitted infections, and tuberculosis.

Worldwide, needle and syringe programs (NSPs) have played a pivotal role in curbing the HIV epidemic by providing prevention information along with syringes and condoms and referring clients to testing and treatment. A recent meta-analyses of 12 studies found that exposure to NSPs reduced the likelihood of HIV transmission [17]. Across Kazakhstan, NSPs are the most widespread service available to PWID, with over 137 located in primary health care clinics, AIDS Centers, and Nongovernmental Organizations (NGOs) [2]. Their primary role is to distribute syringes and condoms, and they are usually staffed by a nurse or social worker, with outreach workers to conduct field-based client recruitment and services.

In Central Asia and globally, many barriers prevent PWID from regularly accessing NSPs, including criminalization of drug use, arrest and policing, negative provider attitudes towards PWID, and lack of staff training and knowledge regarding their needs [9, 18–21]. Official reports from Kazakhstan’s Republican AIDS Center show that less than half (47.5%) of PWID attend NSPs in Kazakhstan [22]. Additional organizational challenges include a lack of evidence-based methods for recruiting new clients and a lack of coordination of care linking NSPs to the AIDS Center and other services.

Despite these challenges, Kazakhstan’s NSPs, with community-based locations, outreach staff with strong connections to PWID communities, and ready access to primary care clinics and NGO services, may be an optimal setting to close the gaps in the HIV care continuum for PWID. A differentiation of care approach advocates for shifting HIV services from traditional medical institutions into peer networks and community-based organizations such as NSPs [23]. This may be particularly useful for PWID, given that they may avoid medical institutions due to stigmatization and criminalization [24].

Engaging vulnerable groups such as PWID requires outreach and intensive coordination of care on the part of drug treatment and harm reduction programs [25, 26]. Evidence indicates that these programs can provide an ideal setting for integration of HIV services, specifically those focusing on seeking, testing, and linking PWID to care [9]. Decentralization of HIV care and integration of HIV services into drug treatment and harm reduction programs brings HIV services to those who would otherwise not have access to them, reduces risky behaviors, and improves adherence to services and health outcomes for PWID living with HIV [25–28]. Despite these proven successes, integrated programs are underused because of a lack of resources, staff training, and client motivation, and therefore implementation of this strategy remains limited both globally and in Kazakhstan [20, 29]. This highlights the need for interventions that strengthen the capacity of Kazakhstan’s NSPs to recruit and test at-risk PWID for HIV, and strengthen their protocols to link or re-link HIV-positive PWID to care.

Finally, there is little implementation research on NSP service delivery in Kazakhstan and Central Asia, with existing reports limited to examining the characteristics of their clients, and their role as stand-alone harm reduction interventions [30]. As HIV testing, linkage and treatment programs rely on a large number of local organizations and government regulations, implementation research is necessary to assess how to deliver interventions in low-threshold real-world NSP settings and what strategies are necessary to optimize and sustain them.

This paper describes the study protocols for evaluating the effectiveness and implementation of Bridge, a three-component HIV intervention, which integrates peer network-based recruitment of PWID to NSPs, rapid testing in NSPs, and linkage from NSPs to treatment and other services through an enhanced Antiretroviral Treatment and Access to Services (ARTAS) case management program [31]. The study uses a stepped-wedge clustered trial in 24 NSPs in Kazakhstan over a 3-year period from 2017 to 2020. Bridge trains health care providers and outreach workers to collaborate to recruit PWID, provide rapid testing, and link them to HIV care, using a package of implementation strategies that include standardized training, supervision, a community of practice, and technical assistance. The study also identifies structural, community, organizational, and client factors that facilitate or impede the uptake and fidelity of Bridge in NSP settings. The design of Bridge responds to calls for differentiated approaches to HIV care and integration of services into harm reduction programs, with implementation strategies tailored to challenges faced by regional NSPs.

## Methods

### Overview of study and study aims

**Fig. 2:**
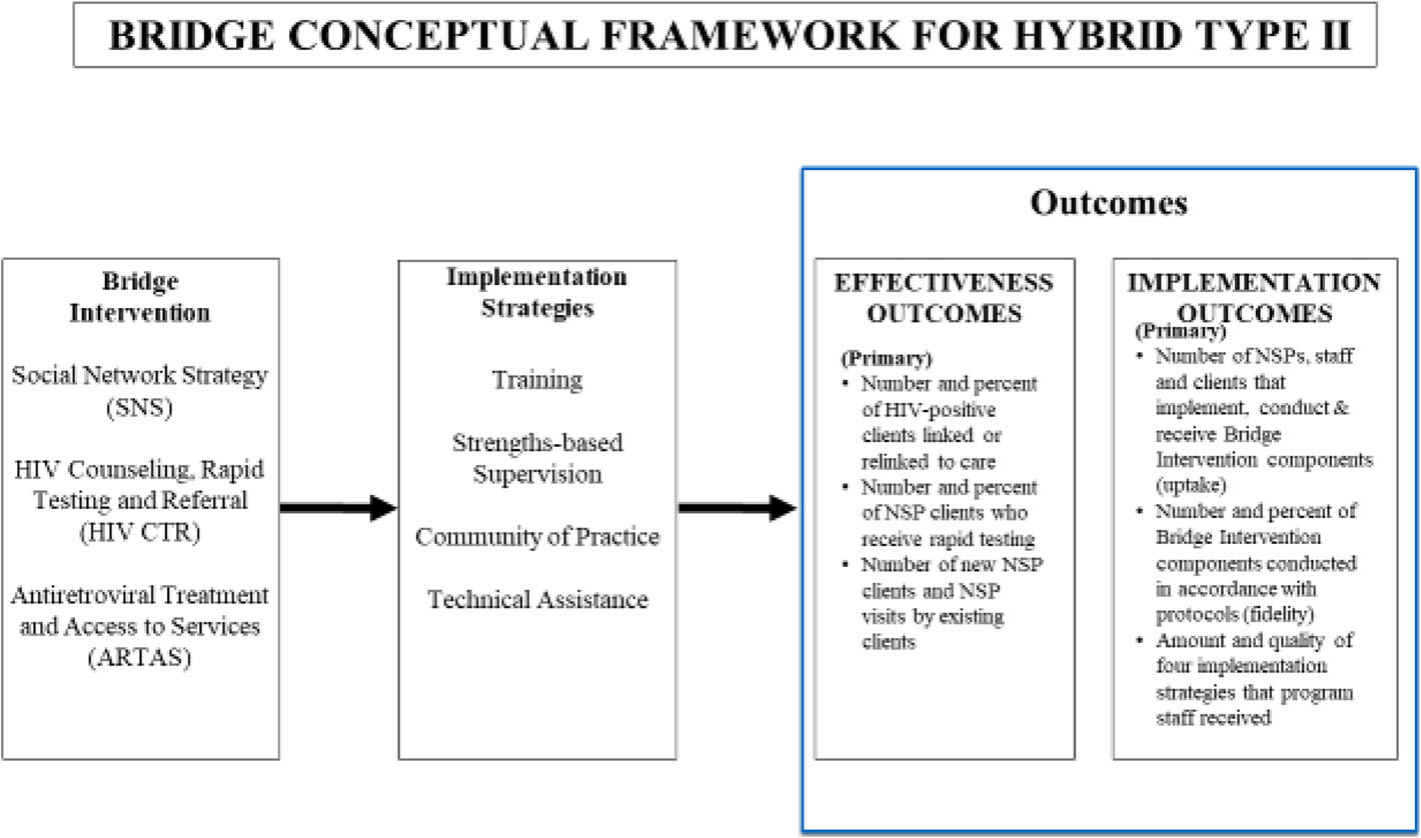
Bridge conceptual framework

Bridge is a Hybrid Type II study [32], which tests effectiveness and implementation outcomes simultaneously to provide evidence supporting the successful delivery of the Bridge intervention in NSPs. Both effectiveness aims and implementation aims have equal weight in our Bridge conceptual model (see Fig. 2).

Primary study aims include:

Aim 1: To evaluate the effectiveness of Bridge on (1) increasing the number of PWID who attend NSPs, (2) increasing the number of PWID who receive a rapid test for HIV at the NSP, and (3) linking HIV-positive PWID at NSPs (including both new cases and those who have not attended HIV care in the past 6 months) to HIV care at the AIDS Center (Effectiveness).

Aim 2: To assess how the Bridge intervention’s package of four implementation strategies (including training, supervision, community of practice, and technical assistance) impacts intervention uptake and fidelity and the intervention effectiveness as described in Aim 1 (Implementation).

Secondary study aims include:

Aim 3: To evaluate the effectiveness of Bridge on (1) increasing retention in HIV care, (2) increasing retention in NSPs, (3) initiating ART, and (4) increasing adherence to HIV treatment regimens and viral suppression (Effectiveness).

Aim 4: To assess how multi-level theory-driven factors (individual, staff, agency, community, structural) influence the implementation and effectiveness of Bridge on primary and secondary effectiveness aims using mixed methods (Implementation).

Aim 5: To estimate the cost of the Bridge intervention and assess implications for feasibility of program expansion and sustainability (Implementation).

### Study design

#### Stepped-wedge study design

**Fig. 3:**
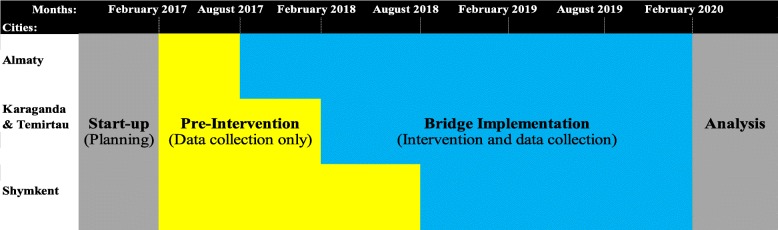
Bridge stepped-wedge design

We utilize a stepped-wedge, cluster trial in three cities containing eight NSPs each, 24 NSPs in total (see Fig. 3). After 6 months of pre-implementation data collection across all study sites, the intervention is implemented in city 1, followed by city 2 6 months later, and city 3 6 months after that. The stepped-wedge design allows us to capture and control for exogenous time-specific effects in our analyses, such as new HIV initiatives or funding shifts. As an alternative to a randomized controlled trial, the stepped-wedge design ensures that all study sites will eventually receive the intervention, addressing an important ethical consideration.

#### Site selection for stepped-wedge

**Fig. 4:**
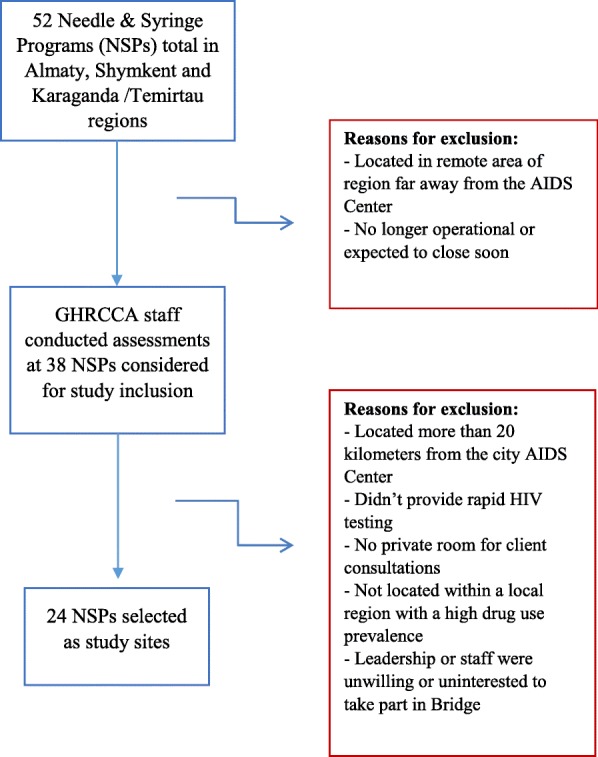
Bridge site selection process

Due to their high prevalence of PWID, sufficient numbers of NSPs and estimated numbers of HIV-positive PWID who remained unlinked to care, we selected the cities of Almaty, Shymkent, and Karaganda/Temirtau (considered as a single city given their geographic proximity and shared administrative oversight). To select eight sites within each city, we carried out a selection via multiple steps depicted in Fig. 4. This process culminated in the selection of 24 NSPs that meet the following criteria: (1) are located within 20 km of the city AIDS Center, (2) provide rapid HIV testing as part of regular services, (3) have a private room available for confidential consultations, (4) are located within regions that have a high number of PWID (as estimated by local AIDS Centers), and (5) where leadership and staff expressed willingness to take part in the study.

#### Research and intervention participants

The study team includes US and Kazakhstan researchers, and program staff based in the 24 NSPs. The study is conducted in close collaboration with NSP leadership, including local and national AIDS Centers and Departments of Health. A Community Advisory Board (CAB) was assembled in each study city, consisting of NSP and AIDS Center leaders, drug treatment clinic leaders, local NGOs, international organizations, and representatives from local associations of people living with HIV. CAB meetings take place twice a year to elicit feedback on study design and implementation challenges and to share findings with these key stakeholders.

##### Bridge program staff

The primary implementers of the Bridge intervention are 24 nurses or social workers (one per NSP) and 48 outreach workers (two per NSP). Organizational leadership selected which staff members would work part-time as implementers of Bridge and receive a supplemental salary for time spent on intervention activities. All Bridge program staff receive training in human subject protection as well as intervention protocols. Bridge staff are both implementers of the intervention as well as research subjects, as described below.

##### PWID participants

We collect data on existing clients of NSPs and AIDS Centers. While we introduce an enhanced recruitment technique (Social Network Strategy, described below) as part of the Bridge intervention, the study does not utilize its own cohort of participants, and instead relies on data collected on existing clients. NSP nurses and social workers obtain verbal informed consent from clients before data collection begins.

All research activities involving human subjects received approval from the Institutional Research Board at Columbia University and the ethics committee of the Kazakhstan School of Public Health.

### Bridge: intervention and implementation strategies

#### Intervention and delivery

Bridge is an integrated intervention which combines (1) Social Network Strategy (SNS), a peer-driven recruitment approach based on social network theory [33, 34], which has demonstrated effectiveness in reaching hidden populations and with HIV testing; (2) HIV counseling, rapid testing, and referral conducted in accordance with both international (WHO, CDC) guidelines as well as national guidelines and protocols; and (3) enhanced Antiretroviral Treatment and Access to Services (ARTAS), an evidence-based case-management intervention to link recently-diagnosed HIV-positive PWID to care, to relink those who have dropped out of care, and to support treatment adherence [31]. Trained staff at each NSP, consisting of a nurse or social worker and two outreach workers, deliver the Bridge intervention. By combining these three evidence-based approaches into a single intervention, Bridge presents a streamlined continuum of care approach to identification, testing, and linkage to care.

#### Implementation strategies

The following four implementation strategies for Bridge are standard across all three cities:

##### Training of Bridge program staff

Nurses, social workers, and outreach workers from each study NSP receive intensive training on Bridge protocols in the month prior to implementation in their city. All Bridge program staff complete a 7-day group training led by the study team. Using an adult learning design guided by social learning theory, staff are trained on how to conduct the program protocols specific to their role. Training includes review of HIV facts, pre- and post-test rapid HIV counseling, how to conduct referrals to the AIDS Center, drug treatment clinics, and medication-assisted treatment and other services. Nurses and social workers are trained in communication skills grounded in Motivational Interviewing [35] to enhance patient engagement and care management and screening tools for mental health and substance abuse. Staff receive intervention manuals, monitoring tools, and all necessary materials for conducting the intervention.

##### Supervision

The Bridge supervision model enhances existing support structures with new methods, resulting in a strength-based team approach for program implementation. To build local capacity and ownership, each local AIDS Center selected a single senior staff member as a program supervisor for all eight sites. The program supervisor is responsible for providing feedback and guidance to NSP nurses and social workers on HIV testing and ARTAS components, and conducting the Community of Practice described below. NSP nurses and social workers supervise outreach workers on the SNS component of Bridge. Supervision protocols promote a collaborative and group learning approach. Staff are encouraged to troubleshoot problems and challenges as a team prior to requesting technical assistance from the research team and to work together to ensure a streamlined experience for new clients.

##### Community of Practice

Bridge utilizes shared learning through a Community of Practice in each city. This takes two forms: a monthly in-person meeting for all Bridge study staff within a site, facilitated by the program supervisor, and an ongoing text-messaging group on WhatsApp, a commonly-used smartphone program. Both forms provide a forum for nurses and outreach workers to discuss ongoing challenges and successes with Bridge implementation, and share new experience and lessons learned.

##### Technical assistance

The Bridge research team responds to technical assistance requests such as troubleshooting problems with assessment instruments or intervention materials, requests for additional training or information on core elements, or requests for guidance in challenging clinical situations. All technical assistance requests and responses are tracked by the research team to inform implementation aims and cost estimates.

### Assessments

In order to capture outcomes related to all the effectiveness and implementation aims described above, the following assessment tools are utilized (Table 1):

**Table 1:** Bridge study outcomes and assessments

Outcome	Description	Assessment tool	Timing of assessment
Study Aim 1: To evaluate, at both client and NSP levels, the effectiveness of Bridge on (1) increasing the number of PWID clients who attend NSPs, (2) on increasing the number of PWID clients who receive a rapid test for HIV at the NSP, and (3) in linking HIV-positive PWID at NSPs (including both new cases and those who have not attended HIV care in the past 6 months) to HIV care at the AIDS Center.			
Linkage to care	The number and percent of HIV-positive clients who visit HIV clinic at least once in the past 6 months	Google-based data collection tool completed by NSP and AIDS Center nurses; AC chart review	Ongoing during both pre-implementation and program implementation periods
HIV testing	The number and percent of NSP clients who have received an HIV test in the past 3 months at the NSP	Google-based data collection tool completed by NSP and AIDS Center nurses; AC chart review	Ongoing during both pre-implementation and program implementation periods
NSP visits	The number of total clients per NSP, number of new NSP clients enrolled in the past 6 months	Google-based data collection tool completed by NSP and AIDS Center nurses; AC chart review	Ongoing during both pre-implementation and program implementation periods
Study Aim 2: To assess how the Bridge intervention’s package of implementation strategies (including training, supervision, a community of practice and technical assistance) impacts intervention uptake and fidelity and the intervention effectiveness outcomes.			
Uptake	The number of NSPs, NSP staff, and NSP clients implementing, conducting, and receiving each component of the Bridge program	Bridge program documentation completed by NSP staff (program activity checklists and reports; Google-based data collection tool) completed by NSP and AIDS Center nurses	Ongoing during program implementation period
Fidelity	The number and percent of Bridge program activities conducted in accordance with the protocols	Bridge program documentation completed by NSP staff (supervision checklists and reports); Community of Practice (CoP) meeting minutes	Ongoing during program implementation period
Dosage and content of training on Bridge components	Amount and quality of training (per Bridge program component) that program staff were exposed to and received	Training attendance records; number of audio recordings and feedback sessions conducted; pre and post training evaluations	Ongoing during program implementation period
Dosage and content of supervision	Amount and quality of supervision that was provided by program supervisors and received by program staff.	Number, frequency, and modality (in-person, by phone, etc.) of supervision sessions held between program supervisor and NSP staff Staff perceptions of supervision	Ongoing during program implementation period
Dosage and content of technical assistance (TA)	Amount and categories of technical assistance provided by GHRCCA staff to study team	Number, frequency and content of technical assistance requests	Ongoing during program implementation period
Dosage and content of Bridge Community of Practice (CoP)	Amount and quality of CoP sessions	Number and content of CoP meetings, participation in CoP, surveys on perceived usefulness of CoP	Ongoing during program implementation period
Study Aim 3: To evaluate the effectiveness of Bridge on (1) increasing retention in HIV care, (2) increasing retention in NSPs, (3) initiating ART, and (4) increasing adherence to HIV treatment regimens and viral suppression.			
Retention in HIV care	The number and percent of HIV-positive PWID who have visited the AIDS Center at least once in the past 6 months	Google-based data collection tool completed by NSP and AIDS Center nurses; AC chart review	Ongoing during both pre-implementation and program implementation periods
Retention in NSPs	The number and percent of NSP clients who have visited the NSP at least once in the past 3 months	Google-based data collection tool completed by NSP and AIDS Center nurses	Ongoing during both pre-implementation and program implementation periods
Enrollment in ART	The number and percent of HIV + PWID (new cases and those who have fallen out of care) who have initiated ART in the past 6 months	Google-based data collection tool completed by NSP and AIDS Center nurses; AC chart review	Ongoing during both pre-implementation and program implementation periods
Adherence to ART	The number and percent of HIV-positive PWID who have taken ART medication as prescribed in the past 6 months	Google-based data collection tool completed by NSP and AIDS Center nurses; AC chart review,	Ongoing during both pre-implementation and program implementation periods
Viral suppression	The number of HIV + PWID with undetectable viral load	Laboratory assay	Ongoing during both pre-implementation and program implementation periods
Study Aim 4: To assess how multi-level theory-driven factors (individual, staff, agency, community, structural) influence the implementation and effectiveness of Bridge on primary and secondary effectiveness aims using mixed methods.			
Multi-level theory-driven factors (client, staff, agency, community, structural)	Knowledge, attitudes, beliefs and experiences of clients, program staff and leadership	Surveys including TCU-ORC, ORCA, PCIS, and other standardized and innovative assessments based on the CFIR model	Once every 6 months (January and July) during both pre-implementation and program implementation periods
Policy and environment monitoring	External events that may influence Bridge program implementation	Record of ongoing policy initiatives and external factors	Ongoing during both pre-implementation and program implementation periods
Study Aim 5: To evaluate the cost-effectiveness of the Bridge intervention, including implications for feasibility of program expansion and sustainability.			
Cost-effectiveness	Costs of implementing individual program elements of Bridge	Records of time spent on program activities and salary of relevant staff	1 month during program implementation period

#### Application-based data collection system

In order to assess effectiveness aims of the study (Aims 1 and 3), point-of-care data entry is conducted at NSPs and AIDS Centers to record services received at each and linkages between the two. Service providers (nurses or social workers) in NSPs and AIDS Centers (nurses) use a Google-based application on a tablet computer to create an electronic case record for each PWID client. Clients receive a random identification number encoded in a QR code on a keychain, which then is scanned to log in and identify each client during regular NSP and AIDS Center visits. NSP nurses or social workers conduct informed consent and enroll clients in this system during their regular visits to the NSP, and then enter data on the services received by that client during visits on the application. As NSPs only serve PWID clients, there is no selection process, and all clients who provide consent are enrolled. A complete list of indicators collected during client visits is included in Table 2.

**Table 2:** Point-of-care service data collected through Google-Based Survey

Data collected at trust point (on services received that visit)			
Materials distributed • Needles/Syringe sets • Needle/Syringe sets for secondary distribution • Condoms	Rapid HIV testing • Test conducted and results? • If reactive, was this a new case of HIV? • Referral for confirmatory testing	Additional referrals • Drug treatment • TB clinics • STI clinics • NGOs • Other	Bridge intervention-specific • Were clients referred by peer recruiter? • ARTAS sessions received and #
Data collected at AIDS Center (on services received that visit)			
Testing services • ELISA or immunoblot confirmatory testing • VL testing • CD4 testing • Testing results delivered to clients	ART • Began, changed, or refilled ART prescription	Additional referrals • Drug treatment • TB clinics • STI clinics • NGOs • Other	

In addition to serving as a data source for primary aims related to testing, linkage to and retention in care, this application measures Bridge intervention uptake, by including indicators to show which, if any, Bridge intervention activities were received that day (see Table 2).

#### Chart review of AIDS Center records

As a second measure of the study’s effectiveness aims, a bi-annual chart review is conducted of AIDS Center records in each study site. This yields a second outcome measure regarding AIDS Center visits (allowing for triangulation of data from multiple sources) and provides records on client care that are often not available at the point-of-care data collection, such as test results, which take up to a week to be processed. Chart review for Bridge utilizes data from the Electronic HIV Case Management System, a government-approved computer system for the collection, storage, transfer, and analysis of epidemiological, laboratory, and clinical data on all registered cases of HIV infection in Kazakhstan. Within this system, a file is created for each client who registers at the AIDS Center. Staff from the epidemiology and treatment departments enter all visits, HIV and STI tests, results and treatment information for each client. Each AIDS Center is responsible for assuring the quality of entered data through regular checks by specialists.

For this study, a summary of AIDS Center client medical records is conducted every 6 months by an AIDS Center staff member trained by the research team. Staff only extract medical record data on PWID clients and enter it into report format that is transferred to the researchers. The data received are de-identified using a code to which only the AIDS Center staff member has access. Indicators entered into the reports include client’s gender and age, whether client has received services from a NSP over the last 6 months and if so, which one, the number of client visits to the AIDS Center over the past 6 months, any CD4 or Viral Load testing done in the last 6 months and results, any initiation or adjustment in the client’s ART regime during the last 6 months, and any sexually transmitted disease testing done during the last 6 months and results.

#### Bridge intervention and implementation strategies delivery data

A number of program materials and reporting forms are used to assess intervention delivery. Nurses and outreach workers fill out monthly reporting forms, which, in addition to being utilized in the supervisory model, provide the primary process measures for the number of Bridge activities completed and a self-assessment of fidelity to the implementation. Reporting forms contain information on the number and category of study activities completed (for SNS and ARTAS), self-reported fidelity to study activities, and narratives on challenges and barriers faced during implementation. Observation of Community of Practice meetings and group text transcript is also used to monitor implementation. Records collected will also inform study aims on assessing feasibility and sustainability.

To evaluate the impact of training, nurses and outreach workers complete pre- and post-training assessments as well as an audio-recorded role-play following the training. Research staff complete documentation related to the number of hours of training delivered to each site.

Tracking technical assistance requests provides valuable information about the challenges faced during implementation. Measures encompass what technological assistance is needed to support implementation, requests for clinical support, content clarification, skills-building, and additional training. Technical assistance data is collected by Bridge staff, who log each request they receive, including by whom, what, where, and how assistance was requested and provided.

#### Staff and leadership surveys

Staff and leadership surveys assess our secondary study aim of examining multi-level factors that impede or facilitate implementation and effectiveness of Bridge. The measurements for this study aim were guided by the Consolidated Framework for Implementation Research (CFIR), which is widely used in implementation research [36]. By examining how these relate to Bridge intervention outcomes at the level of each staff member, NSP, and city, we can assess which factors are associated with successful implementation of the program, as well as control for environmental and contextual changes throughout the implementation period. Nurses, social workers, outreach workers, and the program supervisor complete a staff survey every 6 months. One head doctor or nurse from each NSP, as well as oversight staff at local AIDS Centers, or NGOs complete leadership surveys. Approximately 80 program staff surveys and 30 leadership surveys are completed in each round of surveys, though staff turnover means that different individuals may be included in each iteration.

Survey questions contain measures of selected CFIR constructs. This includes questions about organizational culture, opportunities for growth and learning within the organization, leadership and management styles, attitudes towards HIV, PWID and drug treatment (stigma), challenges faced by each organization and, post-implementation, opinions on the Bridge intervention, acceptability, adoption and appropriateness and effectiveness of the implementation strategies. Surveys are conducted using a computer-assisted self-interview (CASI) program. Participants are compensated $15 for each survey.

#### Policy and environment monitoring

The research team documents contextual changes that are challenging to capture in the staff or leadership surveys, including new protocols or policies regarding HIV, drug use, or other related services, new domestic or international funding sources or HIV-related programming, regularly scheduled activities of the AIDS Center or NSPs that might conflict with Bridge programming, and trends in staff turnover. Research staff in Kazakhstan regularly seek information about such changes and enter this information into a pre-set reporting form. As this data also affects ongoing study activities, it is collected and reviewed weekly by the study team.

#### Cost data

To assess the cost of the intervention, data is collected regarding staff time (including both NSP-based staff and technical assistance from the research team), intervention materials, and compensation for participants. This data collection is conducted after 1 year of implementation in each city, after the intervention is well-established and has reached a steady state. Additional data is gathered on time and materials for providing HIV-related services (HIV rapid testing, confirmatory testing, and ART treatment) at the NSPs and AIDS Centers. Unit costs for these services (staff labor rates and costs of tests and medications) are provided by staff at NSPs and AIDS Centers.

### Quality assurance

Bridge includes a number of quality assurance (QA) protocols to ensure intervention quality and data accuracy. While the primary responsibility for intervention quality assurance remains within the supervisory model, the research team also monitors intervention progress and fidelity on an ongoing basis, through review of supervision records. The research team conducts quarterly observations at each NSP to observe the accuracy and the challenges of a tablet data collection system, and each reporting period, 10% of chart review reports from the AIDS Center are checked for accuracy.

The research team has convened a data safety and monitoring board (DSMB), which consists of Kazakhstan-based researchers. This DSMB meets annually to review ongoing human subjects and data protocols and events.

### Data analysis for primary study aims

Aim 1 Hypothesis: Compared to pre-implementation time blocks, time blocks where Bridge is implemented will have increased numbers of PWID clients who (a) attend NSPs, (b) receive a rapid test for HIV at NSPs, and (c) link to HIV care at the AIDS center, when their test is positive for HIV care in the past 6 months).

Formal hypothesis testing for study aim 1 (the primary effectiveness aim) draws on data from two sources (as shown in Table 1): the Google app-based data collection system and the AIDS Center chart review. The primary test for these hypotheses will be based on patient visits to NSPs and AIDS Centers, compared at the site (NSP) level. This test will utilize a repeated measures approach and will examine differences in outcome measures obtained during the time points before Bridge is implemented in a city (pre-implementation) versus time points that follow the initiation of Bridge intervention implementation (implementation) in the city. We will employ permutation tests (a.k.a. “randomization tests”) for significance testing because of concerns that the distribution of measures may not be approximated well by a small sample size (e.g., *N* = 24 NSPs). Hypothesis testing in this manner involves permuting the Bridge implementation status of each time point; since the time period “assignment” (pre-implementation vs. Bridge intervention implementation) is allocated based on city, permutations will account for clustering by city (i.e., a permutation for NSPs in the same city will all be assigned the same implementation status for that time point). Not only does this conservatively account for shared variance that might be present due to being located in the same city, statistical efficiency can be improved by clustered permutation testing [37]. For inference, the test statistic (difference in means) observed based on actual period assignments is compared vis-à-vis the distribution of test statistics using permuted assignments, with the proportion of permuted test statistics greater than the actual observed taken as the one-sided or half of the two-sided *p* value.

This data requires an analytic approach that accommodates for the non-independence in measures arising from two sources: (1) correlations due to repeated measures with the same NSP and (2) correlations due to NSPs operating in the same city. Hypothesis testing will rely on Generalized Linear Mixed Models (GLMM) and Generalized Estimating Equations (GEE) as described and recommended for stepped-wedge trials [38]. GLMM is exceedingly flexible and powerful but may not be robust to violations of normality assumptions, which will be examined using various descriptive statistics and further can exhibit difficulties in convergence in some cases. GEE is more robust; however, GEE assumes that missing data are missing completely at random while GLMM assumes that data are missing at random. The exact choice will be informed by the results from analyses that shed insight into the extent that various assumptions hold or are violated (e.g., attrition analyses, parameterization fits). In either case, sandwich estimators for variance will be used which are robust to misspecification of the correlation structure [39]. We will use a link function that can properly model the outcome variable as a function of measurement unit or distribution (e.g., logit for binary outcomes, Poisson or Negative Binomial for count and/or rare outcomes).

Sample sizes for effectiveness outcome hypothesis testing (study aims 1 and 3) were determined from power analyses using *α* = 0.05 with two-tailed hypothesis testing and designing the study to have at least 80% statistical power. The smallest effect size will occur for the proportion achieving undetectable viral load; we posited a 25% point increase, which would result in achieving a level of coverage frequently posited to achieve significant reductions in HIV prevalence [40]. Power analyses for this “rate-limiting” effect size used unconditional exact test to simulate permutation testing method of estimating *p* values as well as simulated cluster permutation (i.e., NSPs in a city co-vary together). Results indicate 80% power is achieved with a sample size of 24 NSPs.

Aim 2 Hypothesis: NSPs with greater utilization of the implementation strategies will have increased effectiveness within the time periods of Bridge implementation.

To evaluate the study’s primary implementation aim (study aim 2), we will generate descriptive statistics on Bridge implementation strategies and study activities conducted, including numbers of staffing and clients served. We will use program documentation and supervision forms to generate measures of intervention fidelity, including the percentage of program activities conducted as planned for SNS peer recruiter coaching sessions and ARTAS sessions. These measures will be synthesized into summary measures that will be examined as mediators of effectiveness outcomes. Specifically, we will examine whether these measures are related to variability in effectiveness outcomes across NSPs in the periods of Bridge implementation by adding them as predictors in these effectiveness analyses using the primary outcome assessment data.

#### Cost analyses

We will calculate the estimated net cost of the intervention compared to no intervention and assess implications for feasibility and sustainability by conducting a budget impact analysis. Net cost of the intervention includes the cost of implementing Bridge as well as the cost of providing HIV-related services. Intervention costs will be assessed using micro-costing techniques [41, 42]. A cost for each HIV-related service will be assigned based on the data collected at the NSPs and AIDS Centers. Incremental costs will be calculated using the same approach as the primary outcome, taking into account HIV-related service costs that occur in the absence of the intervention. Budget impact will be calculated separately for the NSPs and the AIDS Centers.

## Discussion

This paper describes the protocols for an implementation study to identify hard-to-reach and stigmatized populations of PWID, test them for HIV, and link them to HIV care, all of which is implemented at existing NSPs in Kazakhstan. This paper also describes how the effectiveness and implementation of this intervention will be rigorously evaluated through multiple assessments in a stepped-wedge, cluster design. This clinical trial has a number of strengths, including the innovative Bridge intervention, a multi-faceted and comprehensive data collective system, a bundle of implementation strategies that enhance existing structures, and integration into local HIV and harm reduction systems.

The Bridge intervention revolutionizes the role that NSPs play in the existing HIV care system in Kazakhstan, transforming them from ordinary harm reduction programs into a single source of support for all stages of the HIV care continuum. It does this through its unique combination of three evidence-based interventions, targeting multiple stages of the HIV continuum of care through a streamlined approach. This integrated approach is strengthened further by involving multiple levels of staff (outreach workers, nurses, social workers, and supervisors). Bridge creatively utilizes a team-based approach and role redefinition to ensure that each site has a set of skilled staff to move clients along the continuum of care and even provides an internal supervision and group learning structure designed to increase local investment and ownership. Finally, by providing program services in NSPs, which are accessible, community-based organizations, Bridge responds to calls for differentiated approaches to HIV care. It takes advantage of existing close relations between outreach workers and local communities of PWID to provide them with linkage and retention support as well as enhanced recruitment and testing services. There are no other programs or services in Kazakhstan that currently provide a comprehensive range of services for PWID at a single location.

As a research study, Bridge provides a range of innovative data collection procedures. Bridge incorporates biological, behavioral, interpersonal, organizational, community and structural level-assessments, allowing for evaluation of intervention effectiveness on multiple levels. Not only are these data collection systems comprehensive, they are also unique and carefully adapted to the context. The tablet-based data collection program is the only system currently being used in Kazakhstan to collect point-of-care data on services and linkages. Finally, Bridge’s comprehensive use of the CFIR framework responds to calls in the literature for a thorough application of CFIR constructs.

By integrating Bridge into existing services in Kazakhstan, this study ensures high external validity, sustainability, and reproducibility to inform the scale up of Bridge in a range of low-threshold settings in Kazakhstan and in other low- and middle-income countries in the future. Bridge was designed specifically for the context of NSPs in Kazakhstan, based off the investigators’ past studies of PWID [43].

### Expected challenges

As an implementation study, Bridge is subject to a unique set of challenges. Because Bridge is deeply integrated into local organizations, it is highly subject to influence from external factors. Unlike a randomized controlled trial, we cannot control changes in funding and staffing in the NSPs, organizational structure in the NSPs or AIDS Center, changes in test systems used, nor can we control for these differences across study sites. The large geographic scale and the long implementation period heighten these risks. Though we cannot control many of these elements, we will rely on staff and leadership surveys and our policy and environment monitoring tools to account for disruptions and control for their influence on the intervention.

Another challenge is the differentiation between the activities related to Bridge and the Bridge study assessments. Some data collection elements, such as the tablet-based data entry system, require a great deal of effort on the part of the NSP staff, and we suspect that our outcomes of feasibility and acceptability of Bridge components might be contaminated by staff reactions to study data collection systems. We have tried to minimize these effects by initiating this data collection system during the pre-implementation period (see Fig. 3). Furthermore, the stepped-wedge design will allow us to measure these effects.

### Implications of Bridge for HIV care among PWID

To our knowledge, this is the only HIV implementation research study conducted with PWID or in harm reduction settings in the Central Asian region to address the full continuum of HIV services from diagnosis of new cases, linkage to HIV care to retention, and adherence to HIV care for viral load suppression. If successful, it may suggest that NSPs and other community-based harm reduction services have a crucial role to play in all stages of the HIV care continuum. Furthermore, our comprehensive implementation measures will show what organizational environments, staff characteristics, and types of implementation strategies and support for NSP staff are necessary for successful implementation an intervention. Moreover, this study informs the scale-up of Bridge intervention to other NSPs in Kazakhstan, as well as in other Central Asian countries and globally to curb the steep rise in HIV rates among PWID. Bridge rigorously examines barriers and facilitators to achieving these goals among the key populations that are so crucial to their success.

## Data Availability

Data collection for this study is ongoing, so no materials are currently available.

## Abbreviations

ART: Antiretroviral therapy
ARTAS: Antiretroviral Treatment and Access to Services
CAB: Community Advisory Board
CoP: Community of Practice
DSMB: Data Safety and Monitoring Board
GEE: Generalized Estimating Equations
GLMM: Generalized Linear Mixed Models
NGO: Non-governmental organization
NSP: Needle and syringe program
PWID: People who inject drugs
SNS: Social Network Strategy

## References

[1] Ganina LY, Yelizaryeva AV, Kaspirova AA, Ivakin VY, Kyukova VA, Abishev AT (2016). Population size estimate of people who inject drugs (PWID) in the Republic of Kazakhstan. Almaty: Ministry of Health and Social Development of the Republic of Kazakhstan, Republican Center for AIDS Prevention and Control.

[2] Kazakhstan Republican AIDS Center. National Report on Achieved Progress in the Implementation of the Global AIDS Response. Almaty: Kazakhstan Republican AIDS Center; 2018.

[3] Baiserkin BC, Abishev AT, Petrenko II, Saparbekov MK, Kazakov CV, Kalynych NF (2017). HIV yesterday, today and tomorrow: implementation of national HIV response measures in the Republic of Kazakhstan.

[4] El-Bassel N, Gilbert L, Terlikbayeva A, Wu E, Beyrer C, Shaw S (2013). HIV among injection drug users and their intimate partners in Almaty, Kazakhstan. AIDS Behav.

[5] Kazakhstan Republican AIDS Center. HIV Surveillance Data. Almaty: Kazakhstan Republican AIDS Center; 2018.

[6] Risher K, Mayer KH, Beyrer C (2015). HIV treatment cascade in MSM, people who inject drugs, and sex workers. Curr Opin HIV AIDS.

[7] Karch DL, Gray KM, Shi J, Hall HI (2016). HIV infection care and viral suppression among people who inject drugs, 28 U.S. jurisdictions, 2012-2013. Open AIDS J.

[8] Mathers BM, Degenhardt L, Ali H, Wiessing L, Hickman M, Mattick RP (2010). HIV prevention, treatment, and care services for people who inject drugs: a systematic review of global, regional, and national coverage. Lancet.

[9] UNAIDS (2017). Ending AIDS: Progress towards the 90–90-90 targets.

[10] Metsch LR, Pereyra M, Messinger S, Del Rio C, Strathdee SA, Anderson-Mahoney P (2008). HIV transmission risk behaviors among HIV-infected persons who are successfully linked to care. Clin Infect Dis.

[11] Loughlin A, Metsch L, Gardner L, Anderson-Mahoney P, Barrigan M, Strathdee S (2004). Provider barriers to prescribing HAART to medically-eligible HIV-infected drug users. AIDS Care.

[12] Malta M, Magnanini MM, Strathdee SA, Bastos FI (2010). Adherence to antiretroviral therapy among HIV-infected drug users: a meta-analysis. AIDS Behav.

[13] Wood E, Hogg RS, Lima VD, Kerr T, Yip B, Marshall BD (2008). Highly active antiretroviral therapy and survival in HIV-infected injection drug users. Jama.

[14] Mathers Bradley M, Degenhardt Louisa, Phillips Benjamin, Wiessing Lucas, Hickman Matthew, Strathdee Steffanie A, Wodak Alex, Panda Samiran, Tyndall Mark, Toufik Abdalla, Mattick Richard P (2008). Global epidemiology of injecting drug use and HIV among people who inject drugs: a systematic review. The Lancet.

[15] Walsh N, Mijch A, Watson K, Wand H, Fairley CK, McNeil J (2014). HIV treatment outcomes among people who inject drugs in Victoria, Australia. BMC Infect Dis.

[16] Wood E, Kerr T, Marshall BDL, Li K, Zhang R, Hogg RS (2009). Longitudinal community plasma HIV-1 RNA concentrations and incidence of HIV-1 among injecting drug users: prospective cohort study. BMJ.

[17] Aspinall EJ, Nambiar D, Goldberg DJ, Hickman M, Weir A, Van Velzen E (2014). Are needle and syringe programmes associated with a reduction in HIV transmission among people who inject drugs: a systematic review and meta-analysis. Int J Epidemiol.

[18] Deryabina A, El-Sadr WM (2017). Uptake of needle and syringe program services in the Kyrgyz Republic: key barriers and facilitators. Drug Alcohol Depend.

[19] Spicer N, Bogdan D, Brugha R, Harmer A, Murzalieva G, Semigina T (2011). ‘It’s risky to walk in the city with syringes’: understanding access to HIV/AIDS services for injecting drug users in the former Soviet Union countries of Ukraine and Kyrgyzstan. Glob Health.

[20] Mills SFC (2015). HIV Cascade Framework. Linkages.

[21] Boltaev AA, El-Bassel N, Deryabina AP, Terlikbaeva A, Gilbert L, Hunt T (2013). Scaling up HIV prevention efforts targeting people who inject drugs in Central Asia: a review of key challenges and ways forward. Drug Alcohol Depend.

[22] Kazakhstan Republican AIDS Center (2016). AIDS Center Activities 2015.

[23] Grimsrud A, Bygrave H, Doherty M, Ehrenkranz P, Ellman T, Ferris R (2016). Reimagining HIV service delivery: the role of differentiated care from prevention to suppression. J Int AIDS Soc.

[24] Macdonald V, Verster A, Baggaley R (2017). A call for differentiated approaches to delivering HIV services to key populations. J Int AIDS Soc.

[25] Altice FL, Bruce RD, Lucas GM, Lum PJ, Korthuis PT, Flanigan TP (2011). HIV treatment outcomes among HIV-infected, opioid-dependent patients receiving buprenorphine/naloxone treatment within HIV clinical care settings: results from a multisite study. J Acquir Immune Defic Syndr.

[26] Berg KM, Litwin A, Li X, Heo M, Arnsten JH (2011). Directly observed antiretroviral therapy improves adherence and viral load in drug users attending methadone maintenance clinics: a randomized controlled trial. Drug Alcohol Depend.

[27] Selwyn PA, Budner NS, Wasserman WC, Arno PS (1993). Utilization of on-site primary care services by HIV-seropositive and seronegative drug users in a methadone maintenance program. Public Health Rep.

[28] Volkow ND, Montaner J (2011). The urgency of providing comprehensive and integrated treatment for substance abusers with HIV. Health Aff (Project Hope).

[29] Volkow ND, Montaner J (2010). Enhanced HIV testing, treatment, and support for HIV-infected substance users. J Am Med Assoc.

[30] Deryabina AP. An assessment of needle and syringe program (NSP) delivery models for people who inject drugs in the Kyrgyz Republic. New York: ICAP; 2016.

[31] Gardner LI, Metsch LR, Anderson-Mahoney P, Loughlin AM, del Rio C, Strathdee S (2005). Efficacy of a brief case management intervention to link recently diagnosed HIV-infected persons to care. AIDS.

[32] Curran GM, Bauer M, Mittman B, Pyne JM, Stetler C (2012). Effectiveness-implementation hybrid designs: combining elements of clinical effectiveness and implementation research to enhance public health impact. Med Care.

[33] Hinde RA (1976). Interactions, Relationships and Social Structure. Man.

[34] Kimbrough LW, Fisher HE, Jones KT, Johnson W, Thadiparthi S, Dooley S (2009). Accessing social networks with high rates of undiagnosed HIV infection: the social networks demonstration project. Am J Public Health.

[35] Miller W, Rollnick S (2012). Motivational interviewing.

[36] Damschroder LJ, Aron DC, Keith RE, Kirsh SR, Alexander JA, Lowery JC (2009). Fostering implementation of health services research findings into practice: a consolidated framework for advancing implementation science. Implement Sci.

[37] Stedman MR, Lew RA, Losina E, Gagnon DR, Solomon DH, Brookhart MA (2012). A comparison of statistical approaches for physician-randomized trials with survival outcomes. Contemp Clin Trials.

[38] Hussey MA, Hughes JP (2007). Design and analysis of stepped wedge cluster randomized trials. Contemp Clin Trials.

[39] MacKinnon JG, White H (1985). Some heteroskedasticity-consistent covariance matrix estimators with improved finite sample properties. J Econom.

[40] Eaton JW, Johnson LF, Salomon JA, Bärnighausen T, Bendavid E, Bershteyn A (2012). HIV treatment as prevention: systematic comparison of mathematical models of the potential impact of antiretroviral therapy on HIV incidence in South Africa. PLoS Med.

[41] Frick KD (2009). Microcosting quantity data collection methods. Med Care.

[42] Neumann P, Sanders G, Russell L, Siegel J, Ganiats T (2016). Cost-effectiveness in health and medicine.

[43] El-Bassel N, Gilbert L, Terlikbayeva A, Beyrer C, Wu E, Chang M (2014). Effects of a couple-based intervention to reduce risks for HIV, HCV, and STIs among drug-involved heterosexual couples in Kazakhstan: a randomized controlled trial. J Acquir Immune Defic Syndr.

